# c-Myc Sustains Pancreatic Cancer Cell Survival and mutp53 Stability through the Mevalonate Pathway

**DOI:** 10.3390/biomedicines10102489

**Published:** 2022-10-05

**Authors:** Maria Anele Romeo, Maria Saveria Gilardini Montani, Andrea Arena, Rossella Benedetti, Gabriella D’Orazi, Mara Cirone

**Affiliations:** 1 Department of Experimental Medicine, Sapienza University of Rome, Viale Regina Elena 324, 00161 Rome, Italy; 2 Department of Research, Advanced Diagnostics, and Technological Innovation, Regina Elena National Cancer Institute, Via Elio Chianesi 53, 00128 Rome, Italy; 3 Department of Neurosciences, Imaging and Clinical Sciences, University G. D’Annunzio, Via dei Vestini 33, 66100 Chieti, Italy

**Keywords:** c-Myc, mutp53, DDR, mevalonate, pancreatic cancer, oxidative stress

## Abstract

It has been shown that wild-type (wt)p53 inhibits oncogene c-Myc while mutant (mut)p53 may transactivate it, with an opposite behavior that frequently occurs in the crosstalk of wt or mutp53 with molecules/pathways promoting carcinogenesis. Even if it has been reported that mutp53 sustains c-Myc, whether c-Myc could in turn influence mutp53 expression remains to be investigated. In this study, we found that pharmacological or genetic inhibition of c-Myc downregulated mutp53, impaired cell survival and increased DNA damage in pancreatic cancer cells. At the molecular level, we observed that c-Myc inhibition reduced the expression of mevalonate kinase (MVK), a molecule belonging to the mevalonate pathway that—according to previous findings—can control mutp53 stability, and thus contributes to cancer cell survival. In conclusion, this study unveils another criminal alliance between oncogenes, such as c-Myc and mutp53, that plays a key role in oncogenesis.

## 1. Introduction

Under physiological conditions, the oncoprotein c-Myc and the tumor suppressor wild-type (wt)p53 establish a negative feedback loop essential to preserve cellular homeostasis. Indeed, wtp53 and c-Myc are able to regulate cell growth and proliferation in an opposite fashion, in a delicate equilibrium that often results in being dysregulated in cancer [1]. Mutations occurring in the genes encoding for both proteins may contribute to the balance being altered between them in cancer cells. Of note, wt and mutant (mut) p53 may behave in an opposite fashion in the activation/inhibition of pro-survival pathways and consequently in the regulation of the processes under their control. For example, while wtp53 inhibits and is inhibited by STAT3 [2,3,4,5,6], mutp53 establishes a positive feedback loop with this pathway, which plays a key role in oncogenesis [7,8]. Interestingly, the mevalonate pathway has been also reported to be inhibited by wtp53 [9] and activated by mutp53 [10,11,12], and the same occurs between thePI3K/AKT/mTOR pathway and mutp53 [13] or wtp53 [14,15]. No exception to this rule exists regarding the relationship that wtp53 or mutp53 may establish with c-Myc. Indeed, wtp53 and its target p21 can inhibit c-Myc [16,17], which in turn inhibits wtp53 activation, through the actions of the c-Myc-inducible long noncoding RNA inactivating P53 (MILIP) [18] while mutp53 sustains c-Myc-dependent rDNA transcription [19]. Moreover, several years ago, it was reported that mutant p53 can transactivate the c-Myc gene through a single-strand DNA or RNA intermediate, and that c-Myc was an important player in mutp53-driven tumorigenicity [20]. Accordingly, in colorectal cancer metastases carrying p53 mutation, c-Myc, free from the negative control exerted by wtp53, was upregulated [21]. Whether c-Myc could in turn influence the expression of mutp53 remains to be elucidated, and this question as well as the underlying mechanism/s involved in such regulation will be addressed in the present study. We unveil for the first time that c-Myc promotes the stability of mutp53 by sustaining the mevalonate pathway in pancreatic cancer cells. The inhibition of the mevalonate pathway has been shown to hamper the interaction between mutp53 and DNAJA1, a protein belonging to HSP40 family, favoring mutp53 proteasomal degradation [11]. HSPs play a key role in sustaining mutp53 stability, and previous studies—including our own— have indicated that targeting chaperones may reduce mutp53 expression, highlighting this as a promising strategy to fight cancer. Indeed, among the numerous consequences of HSP targeting, the reduction in mutp53 is particularly important as it may prevent the oncogenic effects induced by this protein when it undergoes some types of mutations [8,22,23,24,25]. Last but not least, c-Myc is strongly involved in the regulation of DNA damage repair (DDR) both in wt and mutp53 carrying cancer cells, which may have important implication in cancer survival and response to therapies [26], and interestingly, the mevalonate pathway has been also shown to regulate such a response [27]. Therefore, the interconnection between c-Myc, the mevalonate pathway and mutp53 was investigated in this study by inhibiting c-Myc in pancreatic cancer cells. This cancer model was chosen as it is characterized by the fact that p53 mutations more frequently occur in it, compared to other cancers, and by the fact that c-Myc overexpression is a common finding [28].

## 2. Materials and Methods

### 2.1. Cell Cultures and Treatments

PaCa44 cell line (pancreatic ductal adenocarcinoma) (kindly provided by Dr. M. von Bulow University of Mainz, Mainz, Germany) and PT45 cell line (pancreatic ductal adenocarcinoma) (kindly provided by Dr. H. Kalthoff University of Kiel, Kiel, Germany), carrying different p53 mutations (Cys to Ser 176 and Arg to Lys 280, respectively) were maintained in RPMI 1640 supplemented with 10% fetal bovine serum (FBS) (Corning, NY, USA), L-glutamine (100 μg/mL), streptomycin (100 μg/mL) (Aurogene, Rome, Italy) and penicillin (100 U/mL) (Aurogene, Rome, Italy) (complete medium, CM) in 5% CO_2_ at 37 °C. Cells were always detached using Trypsin-EDTA solution (Aurogene, Rome, Italy). Cells were plated in 6-well plates at a density of 2 × 10^5^ cells/well in 2 mL of CM, and the day after, treated with c-Myc inhibitor (i-cMyc) (25 μM and 50 μM) (EMD Millipore, Darmstadt, Germany) for 48 h. In some experiments, cells, plated as described, were treated with the proteasome inhibitor Bortezomib (BZ) (10 nM) (Sigma Aldrich, Burlington, MA, USA) for the last 4 h of treatment. In other experiments, cells, plated as described, were treated with HSP27 inhibitor (i-HSP27) (10 μM) (Medchem express, Monmouth Junction, NJ, USA) and Lovastatin (LOVA) (30 μM) (Sigma Aldrich, Burlington, MA, USA) for 24 h.

To evaluate the role of mevalonate pathways, cells, plated as described, were treated with Mevalonic Acid (20 μM) (Medchem express, Monmouth Junction, NJ, USA) in combination with i-cMyc. The results were evaluated after 48 h of cotreatments. In all experiments, untreated cells were used as control (CT).

### 2.2. Trypan Blue Assay

After treatments, cell viability was evaluated using a trypan blue exclusion assay (Sigma Aldrich, Burlington, MA, USA). Cells were counted by light microscopy using a Neubauer hemocytometer. The experiments were performed in triplicate and repeated at least three times.

### 2.3. c-Myc Silencing

PT45 cells were seeded into 6-well plates at a density of 2 × 10^5^ cells/well and transfected the following day with c-Myc siRNA (si c-Myc, Santa Cruz Biotechnology Inc., Dallas, TX, USA, sc-29226) using INTERFERin^®^ (Polyplus-transfection, Illkirch, France) according to the manufacturer’s instructions. Control siRNA-A (Santa Cruz Biotechnology Inc., Dallas, TX, USA, sc-37007) was used as a scrambled control (scr). After 48 h of transfection, cells were counted by Trypan blue assay and protein expression was evaluated by Western blot analysis.

### 2.4. Western Blot Analysis

After treatments, the cells were washed in PBS 1X, lysed in RIPA buffer (150 mM NaCl, 1% NP-40, 50 mM Tris-HCl (pH 8), 0.5% deoxycholic acid, 0.1% SDS, protease and phosphatase inhibitors) and centrifuged at 14,000× *g* rpm for 20 min at 4 °C. The protein concentration was measured by using the Bio-Rad Protein Assay (Bio-Rad, Hercules, CA, USA) and 15 μg of protein was subjected to electrophoresis on 4–12% NuPage Bis-Tris gels (Life Technologies, Carlsbad, CA, USA), according to the manufacturer’s instruction. The gels were transferred to nitrocellulose membranes (Bio-Rad, Hercules, CA, USA) for 45 min in Tris-glycine buffer and the membranes were blocked in 1 × PBS-0.1% Tween20 solution containing 3% of BSA (SERVA Electrophoresis GmbH, Heidelberg, Germany) incubated with specific antibodies and developed using ECL Blotting Substrate (Advansta, San Jose, CA, USA).

### 2.5. Antibodies

To evaluate proteins expression on Western blot membranes, the following antibodies were used: rabbit polyclonal anti-PARP (1:1000) (Cell Signaling 9542, Danvers, MA, USA), mouse monoclonal anti-p53 (1:100) (clone DO-1, Santa Cruz Biotechnology Inc., Dallas, TX, USA, sc-126), rabbit polyclonal anti-cMyc (1:1000) (Proteintech, 10828-1-AP, Rosemont, IL, USA), rabbit polyclonal anti-HSP70 (1:3000) (Proteintech, Rosemont, IL, USA, 10995-1-AP), rabbit polyclonal anti-HSP90 (1:3000) (Proteintech, Rosemont, IL, USA, 13171-1-AP), rabbit polyclonal anti-HSP27 (1:5000) (Proteintech,18284-1-AP, Rosemont, IL, USA), mouse monoclonal anti-pH2AX (Ser 139) (1:500) (Santa Cruz Biotechnology Inc., Dallas, TX, USA, sc-517348), mouse monoclonal anti-BRCA1 (1:100) (EMD Millipore, Darmstadt, Germany, OP92), mouse monoclonal anti-Ku86 (1:100) (clone B-1, Santa Cruz Biotechnology Inc., Dallas, TX, USA, sc-5280), mouse monoclonal anti-ATM (1:100) (clone G-12, Santa Cruz Biotechnology Inc., Dallas, TX, USA, sc-377293), mouse monoclonal anti-NQO1 (1:100) (clone A 180, Santa Cruz Biotechnology Inc., Dallas, TX, USA, sc-32793) and mouse monoclonal anti-MVK (1:100) (cloneD-3, Santa Cruz Biotechnology Inc., Dallas, TX, USA, sc-390669). Mouse monoclonal anti-β-actin (1:10,000) (Sigma Aldrich, Burlington, MA, USA, A2228) was used as loading control. The goat anti-mouse IgG-HRP (1:10,000) (Bethyl Laboratories, Montgomery, TX, USA, A90-116P) and goat anti-rabbit IgG-HRP (1:10,000) (Bethyl Laboratories, Montgomery, TX, USA, A120-101P) were used as secondary antibodies. The list of antibodies is also reported in Table S1 in Supplementary Materials. All the primary and secondary antibodies were diluted in PBS-0.1% Tween20 solution containing 2% of BSA (SERVA Electrophoresis GmbH, Heidelberg, Germany).

### 2.6. Densitometric Analysis

The quantification of protein bands was performed by densitometric analysis using the Image J software (1.47 version, NIH, Bethesda, MD, USA), which was downloaded from the NIH website (http://imagej.nih.gov, accessed on 1 August 2022).

### 2.7. Measurement of Intracellular Reactive Oxygen Species (ROS)

To measure reactive oxygen species (ROS) production, 10 μM 2,7-dichlorofluorescein diacetate (DCFDA; Sigma-Aldrich, Burlington, MA, USA, D6883) was added to cell cultures for 15 min. Then, cells were detached by Trypsin-EDTA solution (Aurogene, Rome, Italy), washed with PBS 1X and analyzed by FACScalibur flow cytometer (BD Transduction Laboratories, Franklin Lakes, NJ, USA) using CELLQuest Pro-software (version 6.0, BD Biosciences, Franklin Lakes, NJ, USA). For each analysis, 10,000 events were recorded.

### 2.8. Clonogenic Assay

After 48 h of treatment or cotreatment with Mevalonic acid and c-Myc inhibitor, PaCa44 and PT45 cells were detached, plated at low density in 60 mm Petri dishes and grown for twelve days. Surviving colonies were fixed and stained with Cristal Violet (0.5% in methanol) (Sigma Aldrich, Burlington, MA, USA), air-dried and analyzed with Image J. Colony formation was calculated in comparison to untreated control samples, arbitrarily set to 100.

### 2.9. Statistical Analysis

Results are represented by the mean ± standard deviation (S.D.) of at least three independent experiments and statistical analyses were performed with Graphpad Prism^®^ software (Graphpad software Inc., La Jolla, CA, USA). Two-tailed Student’s *t*-test, one-way ANOVA and two-way ANOVA were used to demonstrate statistical significance. Difference was considered as statistically significant when *p*-value was: * <0.05; ** <0.01; *** <0.001 and **** <0.0001.

## 3. Results

### 3.1. Pharmacological or Genetic Inhibition of c-Myc Impairs Pancreatic Cell Survival in Correlation with mutp53 Downregulation

**Figure 1:**
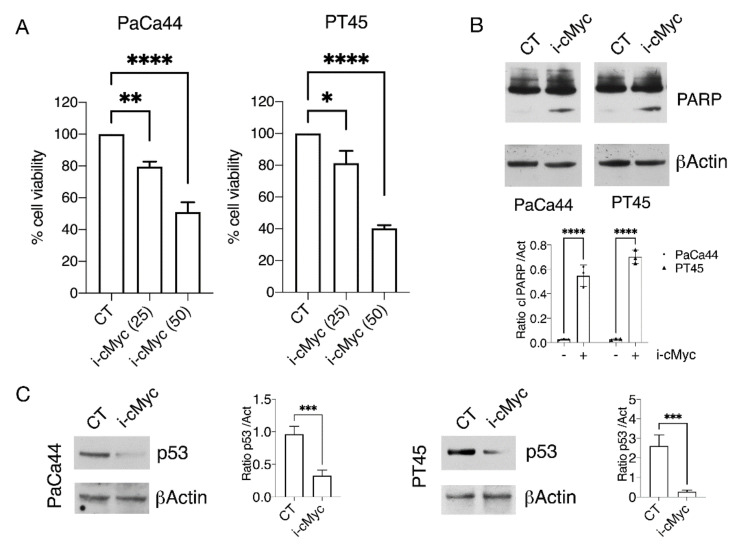

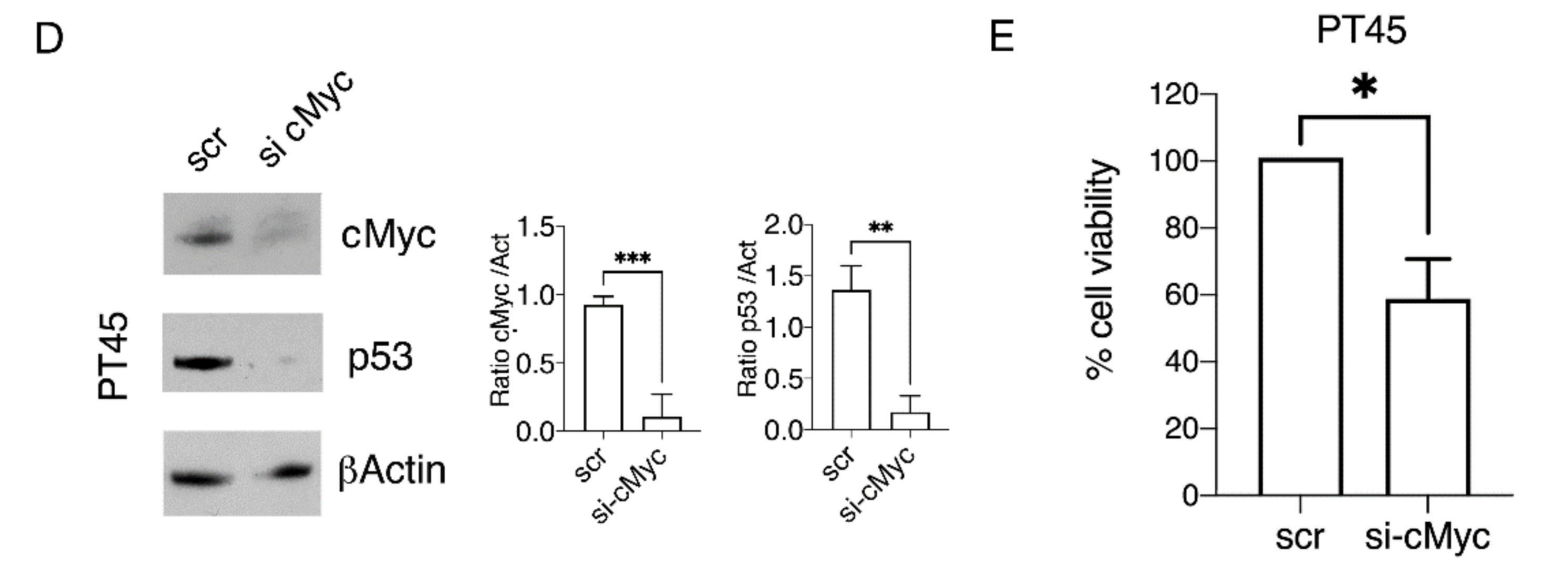
c-Myc inhibition impairs pancreatic cell survival in correlation with mutp53 downregulation. Pancreatic cell lines (PaCa44 and PT45) were treated with two different doses of c-Myc inhibitor (i-cMyc) for 48 h. (**A**) Cell survival was evaluated by trypan blue exclusion assay. The histograms represent the mean plus SD of live cells as percentage of untreated control cells, *p* value: * <0.05; ** <0.01; **** <0.0001; to evaluate cell death (**B**) PARP expression was evaluated by Western blot analyses. βActin was used as loading control and one representative experiment is shown. The histograms represent the mean ± SD of densitometric analysis of the ratio of PARP cl/Act. *p* value: **** <0.0001; (**C**) p53 expression was evaluated by Western blot analyses. βActin was used as loading control and one representative experiment is shown. The histograms represent the mean ± SD of densitometric analysis of the ratio of p53/Act. *p* value: *** <0.001; to evaluate the role of c-Myc in p53 downregulation, PT45 cells were transfected for 48 with cMyc silencing (si cMyc) and control siRNA-A (scr) as control (**D**) protein expression of c-Myc and p53 was evaluated by Western blot analyses. βActin was used as loading control and one representative experiment is shown. The histograms represent the mean ± SD of densitometric analysis of the ratio of cMyc/Act and p53/Act. *p* value: ** <0.01; *** <0.001; (**E**) Cell survival after 48 h of transfection was evaluated by trypan blue exclusion assay. The histograms represent the mean plus SD of live cells as percentage of control cells, *p* value: * <0.05.

We first evaluated the cytotoxic effect of c-Myc inhibition by using a c-Myc inhibitor and found that cell survival of two pancreatic cancer cells, namely PaCa44 and PT45, was impaired in a dose-dependent fashion by such treatment (Figure 1A). Western blot analysis indicated that c-Myc inhibition increased PARP cleavage, suggesting the occurrence of an apoptotic cell death (Figure 1B). Knowing that mutp53 can sustain c-Myc [20], here we explored if the other way around could also occur and found that mutp53 was downregulated in both pancreatic cell lines following c-Myc pharmacological inhibition (Figure 1C). These results were then confirmed by c-Myc silencing (Figure 1D), which reduced both cell survival (Figure 1E) and mutp53 expression level (Figure 1D). Considering that the key role of mutp53 is sustaining pancreatic cell survival [13,24,25,29], these findings suggest that mutp53 downregulation could contribute to the cytotoxic effect induced by c-Myc inhibition in pancreatic cancer cells.

### 3.2. c-Myc Inhibition Promotes the Proteasomal Degradation of mutp53 Independently of HSP Modulation

**Figure 2:**
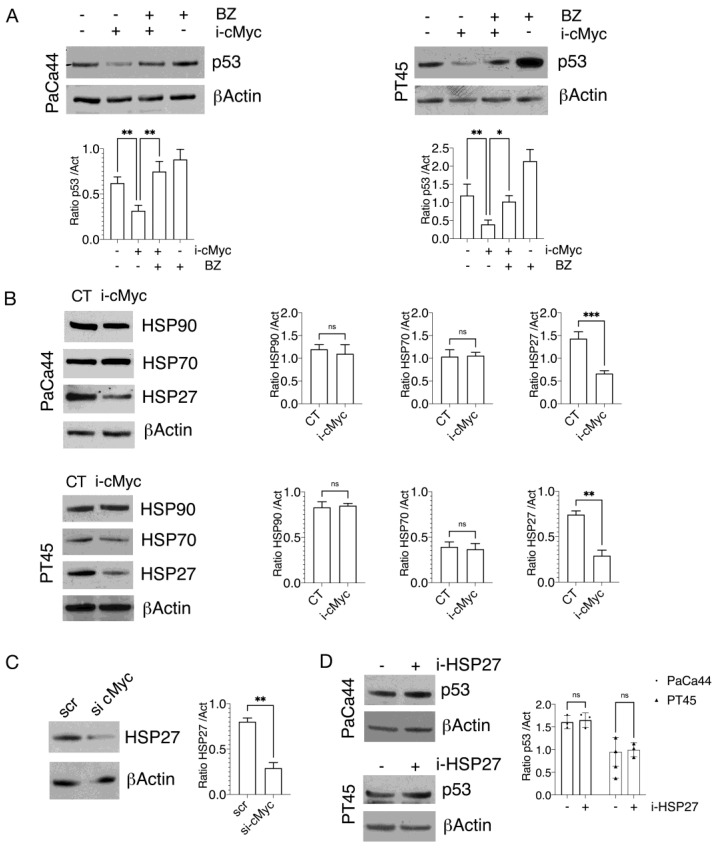
c-Myc inhibition promotes the proteasomal degradation of mutp53 independently of HSP modulation. (**A**) PaCa44 and PT45 cells were treated with cMyc inhibitor (i-cMyc) (50 Μm) for 48 h, in combination or not with Bortezomib (BZ) added for the last 4 h of treatments, and p53 expression was evaluated by western blot analyses. βActin was used as loading control and one representative experiment is shown. The histograms represent the mean ± SD of densitometric analysis of the ratio of p53/Act. *p* value: * <0.05; ** <0.01; (**B**) After 48 h of treatment with c-Myc inhibitor (i-cMyc), HSP90, HPS70 and HSP27 expression were evaluated by Western blot analyses. βActin was used as loading control and one representative experiment is shown. The histograms represent the mean ± SD of densitometric analysis of the ratio of HSP90/Act, HSP70/Act and HSP27/Act. *p* value: ** <0.01; *** <0.001; ns = not significant; (**C**) PT45 cells were transfected for 48 with cMyc silencing (si cMyc) or control siRNA-A (scr) and the expression level of HSP27 was evaluated by Western blot *p* value: ** <0.01 (**D**) PaCa44 and PT45 cell lines treated with a specific HSP27 inhibitor (i-HSP27) for 24 h were assessed for the expression level of p53. βActin was used as loading control and one representative experiment is shown. The histograms represent the mean ± SD of densitometric analysis of the ratio of p53/Act. ns: Not significant.

We then asked whether mutp53 reduction could be due to an increased degradation via proteasome. As shown in Figure 2A, we found that this was the case, as the proteasome inhibitor bortezomib counteracted mutp53 downregulation induced by c-Myc inhibition. Given that HSP90 and HSP70 have been reported to play a key role in regulating mutp53 stability [24,25] and given that the expression of both HSPs has been shown to be influenced by c-Myc activity [30,31], we then asked if c-Myc inhibition could induce mutp53 degradation through HSP70/90 downregulation. As shown in Figure 2B, the expression levels of HSP90 and HSP70 were almost not influenced by c-Myc inhibition, while the small HSP27 was downregulated by such treatment in both PaCa44 and PT45 cells (Figure 2B). This result was confirmed by c-Myc silencing, which reduced the HSP27 expression level (Figure 2C). However, it did not contribute to the reduction in mutp53 stability, as suggested by the use of a HSP27-specific inhibitor that did not influence mutp53 stability (Figure 2D). This indicates that the downregulation of HSP27 was not involved in the reduction in mutp53 expression level induced by the c-Myc inhibitor.

### 3.3. c-Myc Inhibitor Increases DNA Damage in Pancreatic Cancer Cells

**Figure 3:**
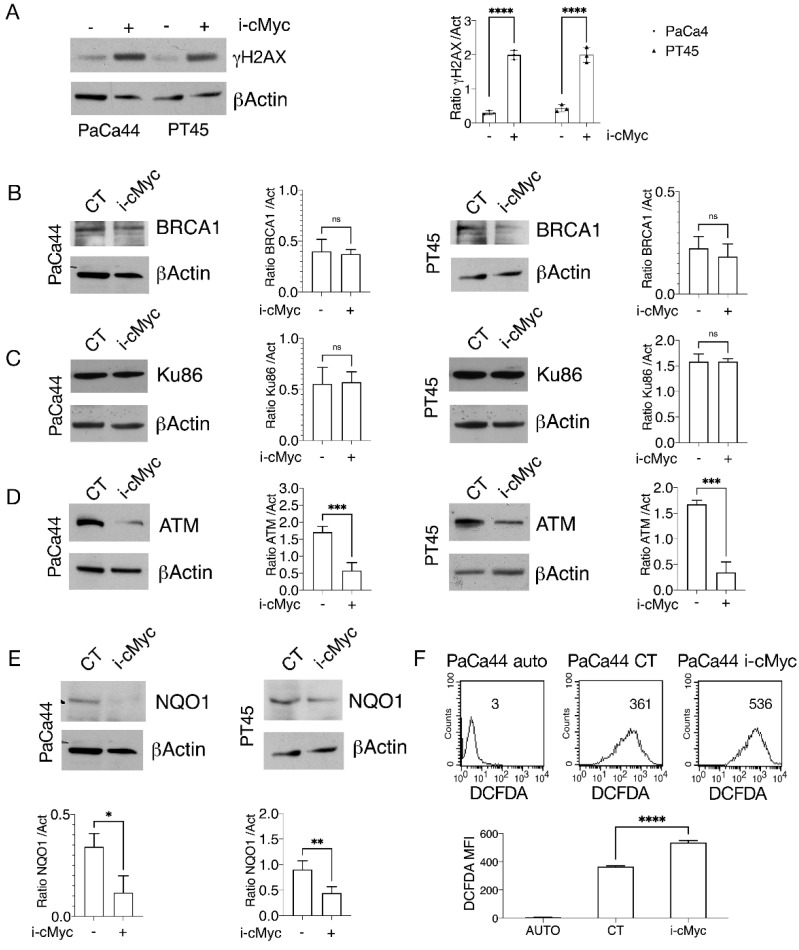
c-Myc inhibition increases DNA damage in pancreatic cancer cells. PaCa44 and PT45 cells were treated with cMyc inhibitor (i-cMyc) (50 μM) for 48 h. (**A**) DNA damage was evaluated studying the phosphorylation of histone H2AX (γH2AX). βActin was used as loading control and one representative experiment is shown. The histograms represent the mean ± SD of densitometric analysis of the ratio of γH2AX /Act. *p* value: **** <0.0001. (**B**) BRCA1, (**C**) Ku86 and (**D**) ATM expression were evaluated by Western blot analyses. βActin was used as loading control and one representative experiment is shown. The histograms represent the mean ± SD of densitometric analysis of the ratio of BRCA1 /Act, Ku86/Act and ATM/Act. *p* value *** <0.001; ns = not significant. (**E**) NQO1 expression was evaluated by Western blot analyses. βActin was used as loading control and one representative experiment is shown. The histograms represent the mean ± SD of densitometric analysis of the ratio of NQO1/Act. *p* value * <0.05; ** <0.01; (**F**) Intracellular ROS level was measured by FACS analysis using DCFDA as staining. Mean of fluorescence intensity (MFI) is indicated. One representative experiment out of three is reported. Auto means autofluorescence of unstained control cells; The bars in the histogram represent the means of MFI of three independent experiments. *p*-value: **** <0.0001.

c-Myc has been reported to affect DDR, essential to repair DNA damage that frequently occurs in highly replicating cancer cells [26,32]. We therefore asked whether the cytotoxic effect mediated by c-Myc inhibition could correlate with increased DNA damage and found that the phosphorylation of H2AX (γH2AX), indicating the occurrence of such effect, increased in both pancreatic cancer cell lines (Figure 3A). Searching for the molecular mechanisms leading to the increase in DNA damage, we evaluated the expression level of DDR proteins involved in both homologous repair (HR) and nonhomologous end-joining (NHEJ) DNA double-strand brake repair. We found that BRCA-1 and Ku86, belonging to HR and NHEJ, respectively, were slightly affected (Figure 3B,C), while ATM, a kinase activated by double-strand DNA brakes and essential to trigger DDR cascade [33], was downregulated (Figure 3D). ATM reduction could therefore contribute to the increase in DNA damage observed in pancreatic cancer cells following c-Myc inhibition. Besides the impairment of DDR, an increase in reactive oxygen species (ROS) and/or the downregulation the antioxidant response could be responsible for increased DNA damage [34]. Therefore, we evaluated whether c-Myc inhibition could modulate NRF2 targets and increase ROS, and found that NQO1, the NRF2 target, was downregulated by such treatment (Figure 3E) and that intracellular ROS increased, as evaluated by DCFDA staining (Figure 3F). These data suggest that both DDR impairment and the downregulation of the antioxidant response could contribute to triggering DNA damage following c-Myc inhibition.

### 3.4. c-Myc Sustains mutp53 Expression Level and Pancreatic Cell Survival through the Mevalonate Pathway

**Figure 4:**
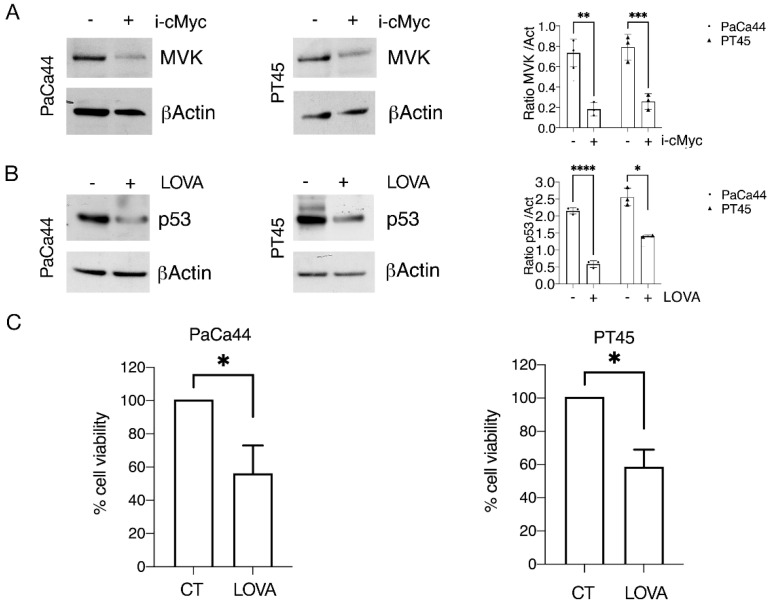
Role of the mevalonate pathway in sustaining mutp53 expression level and pancreatic cell survival. PaCa44 and PT45 cells were treated with cMyc inhibitor (i-cMyc) (50 μM) (**A**) or with Lovastatin (LOVA) (30 μM) (**B**) for 48 h to evaluate mevalonate kinase (MVK) or p53 expression by Western blot analyses. βActin was used as loading control and one representative experiment is shown. The histograms represent the mean ± SD of densitometric analysis of the ratio of MVK/Act and p53/Act. *p* value: * <0.05; ** <0.01; *** <0.001; **** <0.0001; (**C**) cell survival of PaCa44 and PT45 cells treated with LOVA (30 μM) for 48 h was evaluated by trypan blue exclusion assay. The histograms represent the mean plus SD of live cells as percent of untreated control cells, * *p* value: <0.05.

Searching for mechanisms other than HSP reduction that could possibly impair mutp53 stability, we investigated the impact of c-Myc inhibition on the mevalonate pathway in pancreatic cancer cells. This is indeed among the numerous metabolic pathways regulated by c-Myc [35], and it is known to establish a positive feedback loop with mutp53 [12]. We found that the expression of mevalonate kinase (MVK), an enzyme belonging to the mevalonate pathway, was reduced by c-Myc inhibition (Figure 4A), an effect that could contribute to the impairment of mutp53 stability. To investigate if the inhibition of the mevalonate pathway by c-Myc inhibitor could reduce mutp53 expression and mimic the effect induced by c-Myc inhibition, we used Lovastatin, an inhibitor of the mevalonate pathway, and found that it reduced both mutp53 expression level (Figure 4B) and pancreatic cancer cell viability (Figure 4C).

### 3.5. Mevalonate Supplementation Restored mutp53 Expression and Prevented DNA Damage in Pancreatic Cancer Cells Undergoing c-Myc Inhibition

**Figure 5:**
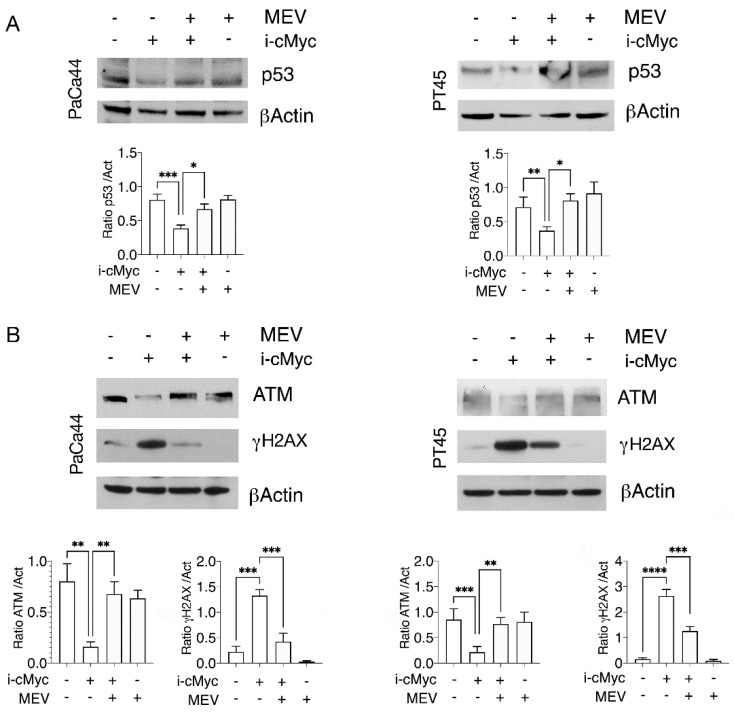
Mevalonate supplementation is able to restore mutp53 expression and to prevent DNA damage in pancreatic cancer cells undergoing c-Myc inhibition. PaCa44 and PT45 cells were treated with cMyc inhibitor (i-cMyc) (50 μM) or MEV (20 μM) or with both for 48 h to evaluate p53 (**A**), ATM and γH2AX (**B**) expression by Western blot analyses. βActin was used as loading control and one representative experiment is shown. The histograms represent the mean ± SD of densitometric analysis of the ratio of p53/Act, ATM/Act and γH2AX /Act; *p* value: * <0.05; ** <0.01; *** <0.001; **** <0.0001.

To further demonstrate the role of mevalonate inhibition in mutp53 downregulation following c-Myc inhibition, we added mevalonate in cells undergoing such treatment and found that such combination prevented mutp53 downregulation in both PaCa44 and PT45 cells (Figure 5A), reinforcing the idea that c-Myc inhibition downregulated mutp53 by interfering with the mevalonate pathway. DNA damage induced by c-Myc inhibition was also reverted by mevalonate supplementation (Figure 5B), and accordingly, the ATM expression level was restored. Altogether, these results suggest that the mevalonate pathway inhibition contributed to mutp53 degradation and to the impairment of DDR induced by the c-Myc inhibitor.

### 3.6. Mevalonate Supplementation Rescued Cell Survival and the Inhibition of Colony Formation Induced by c-Myc Inhibition

**Figure 6:**
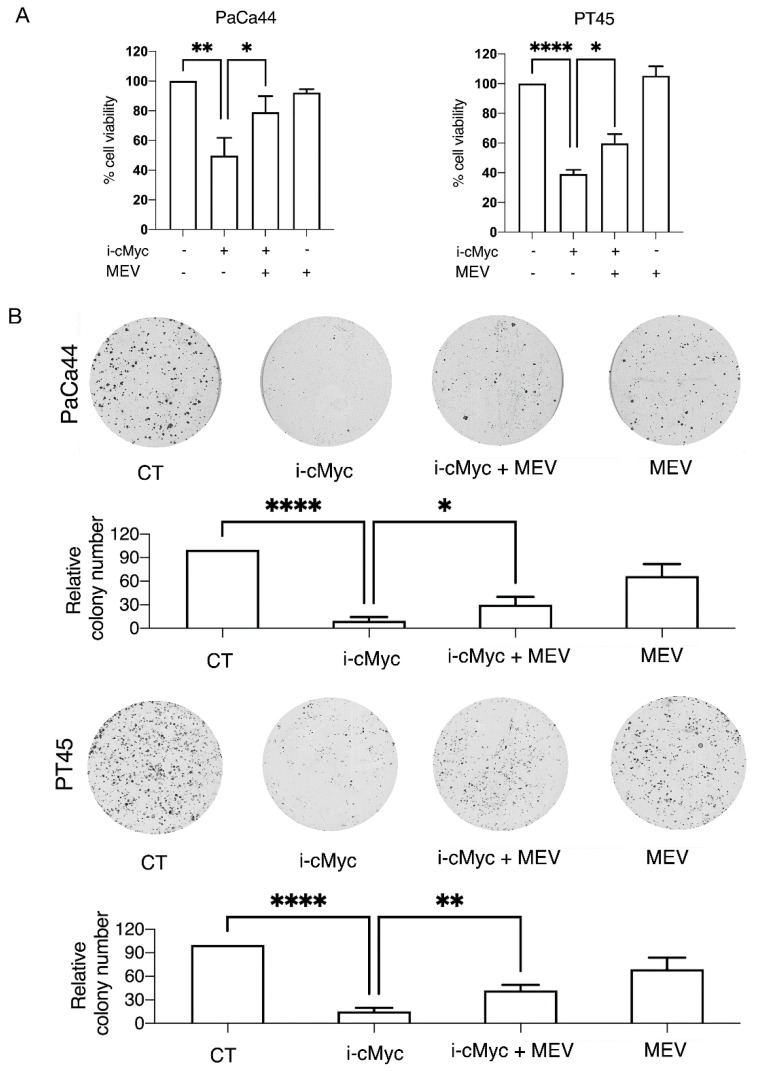
The effects in cell viability and in the colony formation mediated by c-Myc inhibition are counteracted by mevalonate supplementation. PaCa44 and PT45 cells were treated with cMyc inhibitor (i-cMyc) (50 μM) or MEV (20 μM) or with both for 48 h to evaluate cell viability and colony formation. (**A**) Cell survival was evaluated by trypan blue exclusion assay. The histograms represent the mean plus SD of live cells as percentage of untreated control cells, *p* value: * <0.05; ** <0.01; **** <0.0001; (**B**) representative pictures of Paca44 and PT45 colonies stained with crystal violet and histograms of quantitative analyses of colony formation are shown. The numbers of untreated colonies were set to 100. Results are presented as mean ± SD of percent. *p* value: * <0.05; ** <0.01; ****<0.0001.

To evaluate if the mevalonate pathway could contribute to pancreatic cancer cell survival/proliferation in cells treated by c-Myc inhibitor, we assessed cell viability and the capacity to form colonies of these cells undergoing such treatment in the presence or in the absence of mevalonate. As shown in Figure 6A,B, both cell survival and colony formation were reduced by c-Myc inhibitor and restored by mevalonate supplementation in PaCa44 and PT45 cell lines, confirming the role of mevalonate in sustaining pancreatic cell survival, according to its role in mutp53 stability and DNA damage prevention.

## 4. Discussion

Pancreatic cancer is an aggressive cancer characterized by poor response to chemotherapies and whose incidence is continuously growing [36]. Similar to other cancers, c-Myc is frequently overexpressed and strongly drives pancreatic cell growth and proliferation [28,37], A key role in such processes is played by mutp53 [38], given that p53 gene mutations occurring in pancreatic cancer more frequently compared to other cancers may not only lead to the loss of wtp53 functions but also allow for the acquirement of oncogenic properties [12]. These have been reported to be mainly due to several positive feedback loops that mutp53 engages with in pro-oncogenic pathways, the same of which may be instead negatively regulated by wtp53. This is, for example, the case of the mevalonate pathway [9,10,39], which, interestingly, can be included in the huge number of pathways positively regulated by c-Myc [40]. In this study, we show for the first time that c-Myc inhibition negatively affects the mevalonate pathway in pancreatic cancer cells and that this leads to mutp53 downregulation accompanied by an impairment of cell survival. Accordingly, the mevalonate pathway has been previously reported to sustain pancreatic cancer growth and proliferation [41]. Among the molecules most involved in the hyper-stability of mutp53, there are the HSPs, including HSP90 [23,24] and HSP70 [25], whose expression results often upregulated in cancer cells [42]. HSP40 interaction with mutp53, hampered by mevalonate pathway inhibition, was also found to control mutp53 stability [39]. The latter mechanisms may contribute to the downregulation of mutp53 observed in this study, as we found that mutp53 degradation induced by the c-Myc inhibitor correlated with the inhibition of the mevalonate pathway and that HSP70 and HSP90 expression were not affected by c-Myc inhibition. Previous studies have shown that c-Myc is an important player in mutp53-driven oncogenesis [19], that mutp53 can transactivate c-Myc gene [20] and that a positive correlation between the presence of mutp53 and c-Myc overexpression exists in several cancers [21]. Given that c-Myc can be upregulated downstream of STAT3 [43], this pathway could also contribute to c-Myc activation by establishing a positive feedback loop with mutp53 [7,8]. However, as far as we know, this study shows for the first time that c-Myc, through the activation of the mevalonate pathway, sustains mutp53 expression level in pancreatic cancer cells, giving back to this oncosuppressor—becoming an oncogene—the favor to be transactivated by it. This evidence adds another piece to the long and yet-to-be-completed list of the molecules that establish an alliance with mutp53 to promote carcinogenesis. A better understanding of c-Myc and mutp53 crosstalk may offer the possibility to find more effective anticancer strategies able to interrupt the positive feedback loop through which these molecules sustain each other and ultimately drive cancer. The other finding of this study was that c-Myc inhibition, by altering the mevalonate pathway, impaired DDR, reducing the ATM expression level and increasing DNA damage. The latter effect could contribute to the downregulation of the antioxidant response, which can also be affected by c-Myc, according to previous studies showing that c-Myc may activate NRF2 [44] and that the inhibition of the mevalonate pathway can induce oxidative stress [45].

## 5. Conclusions

In conclusion, this study suggests that c-Myc can sustain mutp53 expression through the mevalonate pathway, and by doing so, maintains DDR and controls the oxidative stress, preventing pancreatic cancer cell death.

## Data Availability

The datasets generated and analyzed during the current study are available from the corresponding author upon reasonable request.

## Supplementary Materials

The following supporting information can be downloaded at: https://www.mdpi.com/article/10.3390/biomedicines10102489/s1, Table S1: List of antibodies.
