# Supplementary material for: c-Myc Sustains Pancreatic Cancer Cell Survival and mutp53 Stability through the Mevalonate Pathway
Source: Biomedicines. 2022 Oct 5;10(10):2489. doi: 10.3390/biomedicines10102489 (PMC9599358; doi:10.3390/biomedicines10102489)
Supplement: Supplementary file 1 [file biomedicines-10-02489-s001.zip › biomedicines-1938681-supplementary.pdf]

biomedicines

MDPI

Supplementary Materials

Table S1. List of antibodies.

Primary Antibodies	Supplier	Species	Type	Diluition	Reference
ATM	Santa Cruz Biotechnology Inc.	Mouse	Monoclonal	1:100	sc-377293
BRCA1	EMD Millipore	Mouse	Monoclonal	1:100	OP92
cMyc	Proteintech	Rabbit	Polyclonal	1:1000	10828-1-AP
HSP27	Proteintech	Rabbit	Polyclonal	1:5000	18284-1-AP
HSP70	Proteintech	Rabbit	Polyclonal	1:3000	10995-1-AP
HSP90	Proteintech	Rabbit	Polyclonal	1:3000	13171-1-AP
Ku86	Santa Cruz Biotechnology Inc.	Mouse	Monoclonal	1:100	sc-5280
MVK	Santa Cruz Biotechnology Inc.	Mouse	Monoclonal	1:100	sc-390669
NQO1	Santa Cruz Biotechnology Inc.	Mouse	Monoclonal	1:100	sc-32793
p53	Santa Cruz Biotechnology Inc.	Mouse	Monoclonal	1:100	sc-126
PARP	Cell Signaling	Rabbit	Polyclonal	1:1000	9542
pH2AX (Ser 139)	Santa Cruz Biotechnology Inc.	Mouse	Monoclonal	1:500	sc-517348
b-actin	Sigma Aldrich	Mouse	Monoclonal	1:10,000	A2228
